# A framework for evaluating island restoration performance: A case study from the Chesapeake Bay

**DOI:** 10.1002/ieam.4437

**Published:** 2021-05-25

**Authors:** Jenny Davis, Paula Whitfield, Danielle Szimanski, Becky R. Golden, Matt Whitbeck, Joe Gailani, Brook Herman, Amanda Tritinger, Sally C. Dillon, Jeffrey King

**Affiliations:** ^1^ National Oceanic and Atmospheric Administration National Centers for Coastal Ocean Science Silver Spring Maryland USA; ^2^ US Army Corps of Engineers Baltimore District Baltimore Maryland USA; ^3^ Maryland Department of Natural Resources Annapolis Maryland USA; ^4^ US Fish and Wildlife Service Chesapeake Marshlands National Wildlife Refuge Complex Cambridge Maryland USA; ^5^ US Army Corps of Engineers Engineer Research and Development Center Vicksburg Mississippi USA

**Keywords:** Beneficial use, Coastal protection, Engineering with Nature, Island restoration, Nature‐Based Solutions

## Abstract

The use of natural habitats for coastal protection (also known as Nature‐Based Solutions or NBS) in place of engineered structures like breakwaters and seawalls can yield a wide range of ecological and economic benefits. Despite these advantages, NBS are not commonly implemented for shoreline protection due to uncertainty over the amount of protection afforded by each unique feature and how protective capacity and ecological benefits are likely to change over time as NBS mature and adapt to changing environmental drivers. Here, we highlight the recent restoration of Swan Island in the Chesapeake Bay, Maryland, USA, and the collaborative approach used to evaluate post‐construction performance, as a framework for quantitative evaluation of NBS projects. At Swan Island, 60 000 cubic yards of dredged sediment were used to elevate and restore the island's footprint with an emphasis on increasing its protective and ecological benefits and long‐term resilience to sea‐level rise. Five entities have leveraged resources to quantify the benefits and efficacy of island restoration by conducting pre‐ and post‐restoration monitoring, which supports the development of an integrated, simulation model that includes three “measured” system parameters: wave height, vegetative biomass, and island profile (i.e., elevations). The model will be used to predict island performance under a range of different system scenarios and used to inform adaptive management options. Results will demonstrate the efficacy of leveraging natural and engineered processes to restore island systems while providing a framework for quantifying NBS. *Integr Environ Assess Manag* 2022;18:42–48. © 2021 The Authors. *Integrated Environmental Assessment and Management* published by Wiley Periodicals LLC on behalf of Society of Environmental Toxicology & Chemistry (SETAC). This article has been contributed to by US Government employees and their work is in the public domain in the USA.

## KEY POINTS

- Evaluations of NBS performance at the scale of the individual project are needed to address uncertainties about when and where natural solutions can afford adequate levels of coastal protection.

- A consistent approach to NBS project evaluation will help to foster confidence in the use of natural approaches.

## INTRODUCTION

Native coastal habitats like dunes, wetlands, reefs, and coastal forests are attributed with the protection of developed upland regions due to their ability to absorb and dampen wave energy and slow the inland transfer of water during flood and storm events (Arkema et al., 2017; Guannel et al., 2016; Narayan et al., 2016; Reguero et al., 2018). Through this protective capacity, natural habitats can help to ameliorate the impacts of coastal hazards like storms and chronic erosion while also providing a wide range of ecological benefits and opportunities for recreation and tourism. These benefits make it desirable to use natural habitats in place of traditional hard structures for coastal protection where possible (Sutton‐Grier et al., 2015). The use of natural habitats for coastal protection involves the alignment of engineering with natural processes, which is the guiding philosophy of the United States Army Corps of Engineers (USACE) Engineering With Nature® (EWN®) initiative (King et al., 2020). When used either alone or in combination with engineered structures, natural habitats are often referred to as Natural and Nature‐Based Features (NNBF) or Nature‐Based Solutions (NBS); both terms are used to emphasize the protection afforded by natural habitats in addition to the environmental and socioeconomic benefits with which they are more commonly associated.

Efforts to quantify the effectiveness of NBS at providing protection have concluded that the presence of natural coastal habitats leads to a significantly reduced risk of storm surge flooding and associated insurance claims during extreme weather events (Narayan et al., 2017; Sun & Carson, 2020), and that the restoration of degraded coastal islands increases their ability to protect against storm surge and waves (Wamsley et al., 2009). These analyses were conducted at broad spatial scales (e.g., whole watersheds or county) by documenting economic damage or modeled storm surge inundation during extreme weather events in localities with and without large expanses of natural lands. This approach illustrates the cumulative benefits of intact natural habitats across large geographies, but such results are not directly applicable at the finer spatial scale of the individual project where cost/benefit decisions regarding project feasibility are made. USACE dredging projects are required by law to use the least costly alternative for dredge material disposal that is consistent with sound engineering and environmental practices (Navigation and Navigable Waters, 2012). NBS projects involving the beneficial use of dredged sediments to create or restore habitat are frequently more costly than other options for dredged material disposal due in large part to the precise placement requirements needed for habitat creation. Quantification of protective and ecosystem service benefits at the scale of the individual project may help to tip the balance in terms of cost/benefit valuation and ultimately increase the frequency with which dredged sediments are used to restore coastal habitats.

Uncertainty over NBS response to environmental drivers has been cited as an additional impediment to their widespread adoption (Möller, 2019). Man‐made structures for coastal protection are built to defined criteria in terms of structure life, maximum wave energy they can sustain, and storm surge height they can effectively defend against. In contrast, performance criteria are not easily quantified for NBS and may change over time as the natural components adapt to changing environmental conditions. This ability to adapt to changing environmental drivers can be beneficial. NBS are capable of adjusting their position in the tidal frame in response to changes in sea‐level rise and can self‐repair after storms (Gittman et al., 2014; Rodriguez et al., 2014), whereas man‐made structures can be rendered obsolete by changes in environmental conditions (Hinkel et al., 2014) and can require expensive repairs when storm related‐damages are sustained. Despite the benefits of adaptability, the dynamic nature of NBS leads to uncertainty regarding how a given feature will perform. Among the unknowns about NBS performance are a lack of empirical data describing how effective and resilient NBS are across a spectrum of environmental settings and storm conditions; how the capacity of NBS to mitigate hazards changes over time as sites mature and environmental drivers change; and availability of guiding principles for monitoring and design.

The key to overcoming barriers to NBS implementation is scientifically defensible data that increase understanding of their performance (Morris et al., 2019). Data‐rich performance evaluations that involve site‐specific characterization of physical (e.g., waves, currents, and sediment movement) and ecological benefits and trade‐offs, protective capacity of the feature in question, and interactions among these parameters are particularly needed.

Toward that goal, we present a framework for performance evaluation used for a recently implemented NBS project at Swan Island, Maryland, USA. The framework involves the combination of three project elements (Multidisciplinary Collaboration, Monitoring and Adaptive Management Planning, and Predictive Modeling), which are described below in the context of their application at Swan Island. Although none of these elements are unique to this project, we suggest that their combination is critical to advancing the science of NBS. We do not report project data here; field data and monitoring results will be the focus of future contributions. The goal of this current effort is to encourage a standardized and comprehensive approach to performance evaluation so that the benefits and implications of NBS are well‐quantified. Widespread use of such practice is the key to broader acceptance of NBS for coastal protection.

## SWAN ISLAND: A CASE STUDY

**Figure 1:**
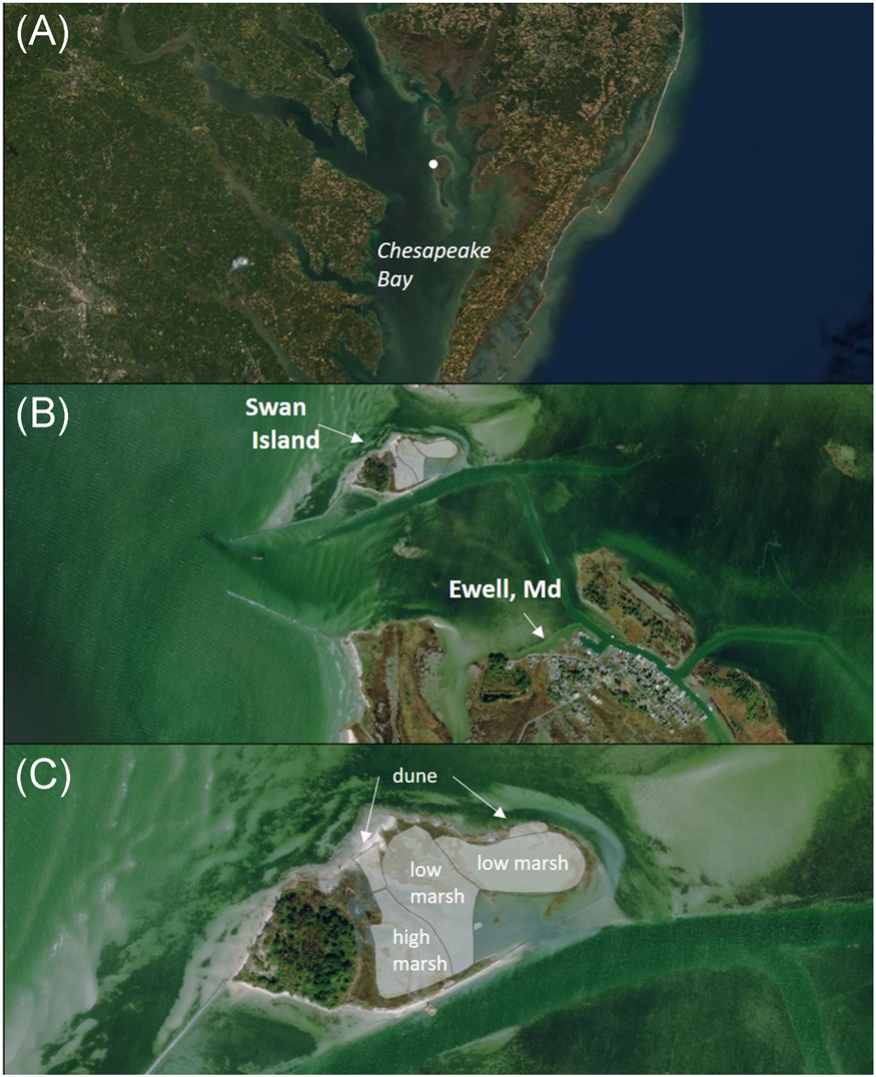
Project location. Swan Island location within the Chesapeake Bay (A), relative to the town of Ewell, Maryland (B) and close‐up with areas of sediment placement highlighted by semitransparent overlay (C)

Swan Island, Maryland, USA, is an uninhabited 25‐acre island within the Martin National Wildlife Refuge in Tangier Sound, Chesapeake Bay. Swan Island is located directly to the north northwest of the town of Ewell, Maryland, and serves as a natural wave break, protecting the shoreline of Ewell from waves generated in the Chesapeake Bay (Figure 1). Like other islands in the mid‐Chesapeake Bay, Swan Island experiences high rates of erosion; its northwest shoreline has been receding at rates of up to 2 m year^−1^ (Perini Management Services, 2014). Land subsidence in the region contributes to elevated rates of relative sea‐level rise (4.5 mm year^−1^) and presents a further challenge to the long‐term survival of these islands by increasing the frequency of wash‐over events and accelerating the conversion of low‐lying marsh habitat to open water (Erwin et al., 2011).

The town of Ewell is only accessible by boat and therefore highly dependent on the federally maintained navigation channel that provides access (Figure 1). To ensure navigability, maintenance dredging occurs approximately every 10 years. In the past, dredged sediments have been deposited in a privately owned, confined disposal facility on nearby Easter Island. As of 2008, this site has been filled to capacity and is not suitable for the placement of additional material without a costly reinforcement of its containment berms.

**Figure 2:**
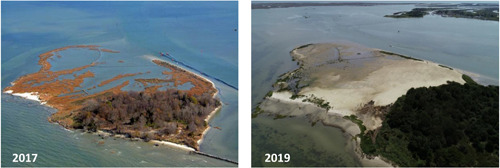
Aerial images of Swan Island before (left) and after (right) sediment placement and planting

During the most recent dredge cycle (October 2018–April 2019), the vulnerability of Swan Island to further degradation and the need to find a new placement option (other than Easter Island) provided the perfect synergy for island restoration through beneficial use of dredged sediments. The restoration plan involved the placement of approximately 60 000 cubic yards of sediment, which were graded to create low dunes and an extensive region of high marsh habitat, and to raise the low marsh by 20 cm on average (Figure 1). The island was transformed from one characterized by low and highly fragmented marsh to one with a wider range of habitats (including low and high elevation marsh and dunes) that sit higher in the tidal frame (Figure 2). The restoration is predicted to have significant benefits in terms of ecosystem service provision, increased resilience of Swan Island to future sea‐level rise, and enhanced ability to protect the shoreline of Ewell from wave energy.

Projects like the Swan Island restoration serve as case studies for EWN principles and provide a valuable opportunity to quantify the habitat trade‐offs, ecological implications, and coastal protection benefits associated with NBS at the scale of the individual project. Although USACE districts across the country have shown increasing interest in NBS as a constructive means of managing dredged sediments, it is rare for such projects to have the funding necessary to support robust evaluation and monitoring. To address this limitation and contribute to a greater scientific understanding, partners from a number of federal and state agencies are committing the resources necessary to evaluate the outcomes of the Swan Island restoration.

## THE SWAN ISLAND PROJECT FRAMEWORK

### Multidisciplinary collaboration

NBS like Swan Island involve the intentional alignment of ecology and engineering for the purpose of shoreline protection. These two disciplines are inextricably linked when it comes to the restoration of coastal ecosystems. As a result, efforts to evaluate their performance require diverse expertise from both fields. Engaging potential partners early in the NBS planning process is critical not only for ensuring that a range of disciplines are represented but also for help in shouldering both the cost and workload. EWN also recommends and prioritizes collaboration as a means of obtaining the best outcomes when pursuing nature‐based strategies. Science‐based collaboration organizes and focuses interests, stakeholders, and partners to reduce social friction, resistance, and project delays while producing more broadly acceptable projects (King et al., 2020).

At a minimum, NBS projects require collaboration among the landowner, dredge professionals, and a partner with expertise in habitat restoration. Swan Island is part of the Martin National Wildlife Refuge and as such is under the stewardship of the United States Fish and Wildlife Service (USFWS). Refuge partners were actively engaged in planning and design for the project, as well as post‐placement quantification of ecosystem services. The navigation channel that provides access to the town of Ewell is maintained by the USACE Baltimore District. Project managers and scientists from USACE Baltimore initiated and funded the dredging project, developed the placement design, conducted stakeholder engagement and environmental coordination, and performed contract administration of the project, working directly with the dredge contractor to ensure optimal results. In addition to these key players, the Swan Island team includes coastal ecologists (Maryland Department of Natural Resources and NOAA's National Centers for Coastal Ocean Science) who are working to quantify the ecological implications of the Swan Island restoration as well as scientists from the USACE Engineering Research and Development Center (ERDC) with expertise in both hydrodynamics and ecological modeling. The project team worked collaboratively to develop a comprehensive monitoring plan that includes collection of field data necessary to evaluate the performance of Swan Island in terms of coastal protection, habitat provision, and changes in performance over time as the site matures.

### Monitoring and adaptive management planning

Through a 2‐day moderated workshop facilitated by the USACE ERDC modeling team, the project team along with a group of invited stakeholders developed a Monitoring and Adaptive Management Plan (MAMP) following the steps previously laid out by Thom (2000) and Grant and Swannack (2008). The MAMP serves as a living document, a blueprint for the project team to document and communicate a shared vision for the project and ensure the process is transferable to other NBS. Primary components of the MAMP include:

#### Clear goal statements

Our agreed‐upon monitoring goals centered around answering the following three questions:

1. How have the restoration actions influenced the capacity of Swan Island to provide protection from wave energy to the town of Ewell?
2. How have the restoration actions influenced the ecosystem services provided by Swan Island?
3. How will the protective capacity and ecosystem services provided by Swan Island be influenced by sea‐level rise?

#### Conceptual model of system

**Figure 3:**
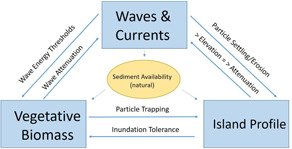
Conceptual model of primary system components defined through a collaborative process as being required to quantify coastal resilience performance of Swan Island

A working hypothesis about how the system in question functions is central to developing an MAMP. Ideally, the development of this hypothesis will involve a wide range of stakeholders of varying expertise and result in an agreed‐upon vision of which components of the system are most critical to its function and how those components interact with each other; in short, a conceptual model. A well‐defined conceptual model provides a map for defining the necessary data to collect during monitoring efforts and can be used to inform the required frequency of data collection efforts. Development of the Swan Island conceptual model was an iterative process involving all members of the project team plus additional stakeholders including local academic, governmental, and non‐profit researchers with expertise in dredging, wetland restoration, and natural resource management. The goal was to include a broad range of expertise for additional perspective. A separate concurrent effort is focused on the perspectives of Smith Island residents. Island residents were not represented in our process so that a manageable workshop size could be maintained. The conceptual model produced through this process highlights the importance of water movement (waves/currents), plant biomass, and elevation (island topography and nearshore bathymetry) as the dominant factors influencing Swan Island's performance and defines the interactions between them (Figure 3).

#### Monitoring plan and decision framework

Clear goal statements and a conceptual model of system performance guide the selection of metrics that must be incorporated into monitoring plans to ensure that project goals are met. In the case of Swan Island, vegetative biomass characteristics (percent cover by species and canopy height), island surface elevation and shoreline position, water movement (waves and currents), and suspended sediment concentration were identified as the core metrics necessary to evaluate changes in the performance of Swan Island in response to restoration efforts (Figure 3). With an agreed‐upon list of minimum metrics in hand, the research team agreed on collection frequencies, methodologies, and data‐sharing procedures. Our monitoring design mirrored those mandated by the National Fish and Wildlife Federation for their National Coastal Resilience Fund projects (NFWF, 2019). In addition to ensuring that the data needs for each of the primary model components were met, we engaged regulatory agencies that have a role in permitting projects similar to Swan Island to ensure that our data collection efforts adequately address their concerns regarding NBS projects. Monitoring efforts began in 2018 before the dredging and restoration activities began and continue with varying frequency as appropriate for each parameter (e.g., vegetative and/or elevation data are collected annually and water level is measured at 5‐min intervals).

Despite the ability of NBS to adapt, they may require maintenance (Mitchell & Bilkovic, 2019), particularly after extreme events that lead to significant changes in biomass density or surface elevation (e.g., ice scour). While we are not aware of available data on the maintenance costs specific to island NBS, previous estimates that consider implementation and maintenance suggest that NBS, in general, are more cost‐effective than traditional engineered structures (Narayan et al., 2016; NOAA, 2020). The extent to which maintenance is required for NBS will vary by site and context and thus is challenging to predict (Seddon et al., 2020). A MAMP addresses this uncertainty by identifying thresholds that will automatically trigger corrective maintenance actions. Such actions are required when changes in the NBS feature are significant enough to result in reduced performance. The specific criteria upon which corrective actions are based will vary by type and location of NBS. For Swan Island, the development of the decision framework was a group effort based on preliminary data and expert opinion about system performance. The ultimate goal of sediment placement at Swan Island was to maintain and enhance the island's capacity to shield the town of Ewell from wave energy. The project team identified island areal extent, elevation, and vegetative cover as the parameters most likely to influence this capacity. As a result, the thresholds that would result in corrective action are centered on changes in these parameters (Table 1). These same parameters are commonly used in MAMPs for marsh restoration projects with the specific performance levels that trigger action calibrated to the system in question (Folse, 2020; Weinstein et al., 1997). There was no regulatory requirement to include adaptive management in the Swan Island project; corrective actions to make sure that project goals are met rely on the voluntary actions of the project team. As with other components of the MAMP, the decision framework is a living document that will be refined throughout the monitoring phase as our understanding of system performance increases.

**Table 1:** Excerpt from Swan Island Decision Framework

Metric	Performance threshold	Adaptive management action
Percent vegetative cover	Percent cover in planted regions increases to 50%–75%	Replant failing areas, consider the use of different species if significant changes in elevation have occurred
Marsh surface elevation	Maintain or increase relative to designed elevations	Additional placement if surface elevations do not maintain range necessary for intended habitat type
Shoreline position	Maintain current shoreline position	Implement living shoreline to protect against further loss, reclaim designed footprint with additional placement

*Note*: Metrics represent data that are collected as part of monitoring program. Performance thresholds indicated conditions that trigger adaptive management actions if not met.

##### Modeling

Natural systems are inherently dynamic in space and time, and respond to environmental drivers that are equally dynamic. To address the inherent uncertainty associated with the long‐term performance of Swan Island, the project team is developing an integrated hydrodynamic and ecological simulation model that will assist in testing assumptions about how the island and its component habitat types will change as the site matures, and in response to changes in environmental drivers (Herman et al., 2020). On‐the‐ground monitoring efforts at Swan Island will provide the data necessary to parameterize and evaluate the simulation model.

When complete, the model will be used to predict changes in Swan Island in response to a range of future sea level and storm scenarios and to quantify the ecosystem services and coastal protection provided by created and restored islands. At Swan Island, the model will be used to examine specific questions: (1) the resilience of the island and its component habitat types to sea‐level rise and storms, (2) how the long‐term performance of Swan Island impacts its capacity to protect the Ewell shoreline from wave energy, and (3) how often additional sediment placement and other maintenance activities may be required to maintain optimal ecological and physical conditions.

The simulation model is a direct extension of the overall project framework. By defining specific goals, conceptualizing the system with respect to those goals, and designing a monitoring program based on the conceptual model, this framework results in the data necessary for a quantitative evaluation of system performance under current and future conditions, and ultimately for valuation of that performance. The project framework presented above, when applied to other NBS projects, will yield the data necessary to evaluate uncertainties in long‐term resilience and protective capacity.

## FUTURE DIRECTIONS

Incorporation of natural habitats into coastal protection strategies can yield diverse benefits that include the provision of habitat for commercially and recreationally important fishery species, water quality remediation, carbon sequestration, and resilience to SLR and other environmental stressors (Barbier, 2013; Engle, 2011). These ecosystem service benefits, combined with their proven ability to attenuate wave energy, store flood waters, and provide a physical barrier to the inland transfer of water, make NBS preferable to traditional engineered structures where conditions allow. Greater acceptance of NBS will not come until decision makers are comfortable that nature‐based approaches can provide adequate levels of protection and not lead to undesirable environmental trade‐offs. A number of pilot projects have been implemented across the United States in recent years (Bridges et al., 2018), and these visual demonstrations of NBS are a critical component of increased acceptance. We propose that the most urgent need for greater acceptance of NBS is greater scientific understanding of performance at the scale of the individual project both in terms of resilience of NBS to local environmental drivers and the protective benefits that each project confers. Multidisciplinary performance analyses that quantify both ecological and engineering outcomes will generate the data necessary to compare the use of NBS for coastal protection with that of traditional engineered structures in terms of their true costs and benefits. Adhering to a prescriptive framework, like the one presented here, will facilitate such analyses while also increasing understanding of how a given NBS is performing, how to plan for future management to correct performance deficiencies, and ultimately will elicit confidence in regulators and build public support for this type of federal investment while providing the data needed to build confidence among private investors as well. The data collected at Swan Island and subsequent performance evaluation and modeling products will be shared among US Army Corps Districts nationwide and distributed broadly among NBS practitioners to promote the optimal design of islands as NBS and to increase acceptance of such practices.

## CONFLICT OF INTEREST

The authors declare that there are no conflicts of interest.
